# Identification of anti-inflammatory and other biological activities of 3-carboxamide, 3-carbohydrazide and ester derivatives of gatifloxacin

**DOI:** 10.1186/1752-153X-7-6

**Published:** 2013-01-14

**Authors:** Najma Sultana, M Saeed Arayne, Asia Naz, M Ahmed Mesaik

**Affiliations:** 1 Department of Pharmaceutical Chemistry, Faculty of Pharmacy, University of Karachi, Karachi, 75270, Pakistan; 2 Department of Chemistry, University of Karachi, Karachi, Pakistan; 3 Dr. Panjwani Center for Molecular Medicine and Drug Research, International Center for Chemical and Biological Sciences, University of Karachi, Karachi, Pakistan

**Keywords:** Gatifloxacin analogues, Oxidative burst response, T-cell proliferation, Fluoroquinolone NSAIDS

## Abstract

**Background:**

Seventeen 1,4-dihydroquinoline-3-carboxamide and 1,4-dihydroquinoline-3-carbohydrazide derivatives of gatifloxacin have been prepared with a facile one step synthesis aiming to improve antibacterial, antifungal and immunological activities. The methodology allows the introduction of a variety of substituents such as amines, alcohol, phenol, amides and alkyl halides into the core structure of gatifloxacin.

**Results:**

The analog N-(3-aminophenyl)-1-cyclopropyl-6-fluoro-8-methoxy-7-(3-methylpiperazin-1-yl)-4-oxo-1,4-dihydroquinoline-3-carboxamide has been identified as a potentially excellent anti-inflammatory agent, which exhibited highly potent effects on the oxidative burst activity of whole blood phagocytes (IC50 <12.5 μg mL^-1^), neutrophils (IC50 <0.1 μg mL^-1^) and macrophages phagocytes (IC50 <3.1 μg mL^-1^) as well as potent T-cell proliferation inhibitory effect (IC50 3.7 μg mL^-1^) while having comparable antibacterial activity to gatifloxacin. Another analog, 1-cyclopropyl-6-fluoro-8-methoxy-7-(3-methylpiperazin-1-yl)-4-oxo-N-phenyl-1,4-dihydroquinoline-3-carbohydrazide has tremendous T-cell proliferation inhibitory effect IC50 <3.1 μg mL^-1^ as compared to prednisolone, whereas, 3,5-dihydroxyphenyl1-cyclopropyl-6-fluoro-8-methoxy-7-(3-methylpiperazin-1-yl)-4-oxo-1,4-dihydroquinoline-3-carboxylate and 2-hydroxyphenyl-1-cyclopropyl-6-fluoro-8-methoxy-7-(3-methylpiperazin-1-yl)-4-oxo-1,4-dihydroquinoline-3-carboxylate envision good inhibitory activity on T-cells proliferation (IC50 6.8 & 8.8 μg mL^-1^ respectively).

**Conclusions:**

The structural modification at carboxylic group has resulted in improved anti-inflammatory activities with comparable antibacterial activity to gatifloxacin. We believe that C3 structural modifications of gatifloxacin are definitely important in bringing major immunomodulatory changes in these compounds.

## Background

An infection is a pathological process whereby an exogenous agent (fungus, bacterium or virus) invades the body causing some form of injurious dysfunction. Upon infection of bacteria, inflammatory cytokines (such as TNF-α) are produced from macrophages and can lead to various conditions such as allergic diseases, autoimmune diseases and inflammatory diseases [1], such as cystic fibrosis characterized by chronic neutrophilic inflammation [2,3]. Such diseases are required to be treated with immune system-harmless anti-inflammatory agents and antimicrobial agents in combination. Fluoroquinolones have long been used as antimicrobial therapy in respiratory, urinary, GI and sexual infections [4]. Several studies have shown that new fluoroquinolones also possess immunomodulatory properties beyond their antimicrobial effects [5]. Gatifloxacin sesquihydrate, a synthetic broad-spectrum antimicrobial fluoroquinolone [6-9], has also an inhibition effect on the production of inflammatory cytokines by macrophages, monocytes or peripheral lymphocytes and particularly suppresses bacterial infection-induced inflammation [10,11]. On the basis of anti-inflammatory activity of gatifloxacin, our initial efforts for new anti-inflammatory agents included the structural modification of gatifloxacin in an anticipation of preservation of the antibacterial activity with enhancement of its anti-inflammatory activity.

It is established that N1, C2–H, C3-carboxylic acid, C4-carbonyl, C6-F, and C7-piperazine are essential or beneficial for the antibacterial activity of fluoroquinolones [12,13]. Modification or elimination of these groups would also give us the valuable structural information for the improvement of anti-inflammatory effects. So far only few lipophilic gatifloxacin analogues are reported in literature [14,15]. Dharmarajan and coworkers prepared sixteen lipophilic N-substituted piperazinyl Mannich bases of gatifloxacin [14]. Similarly Mauro et al., [12] synthesized lipophilic gatifloxacin derivatives and evaluated for their anti-tubercular activity, all analogs proved to be less potent than parent drug.

**Scheme 1:**
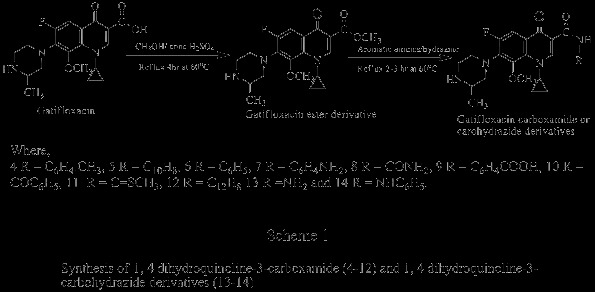
Synthesis of 1, 4 dihydroquinoline-3-carboxamide (4–12) and 1, 4 dihydroquinoline-3-carbohydrazide derivatives (13–14).

**Scheme 2:**
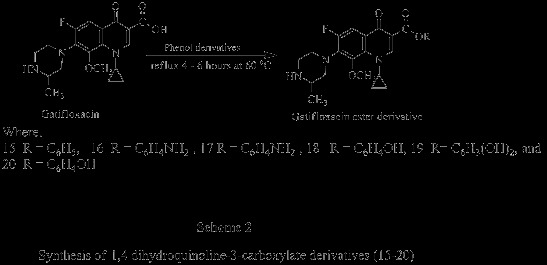
Synthesis of 1,4 dihydroquinoline-3-carboxylate derivatives (15–20).

We prepared seventeen different derivatives of gatifloxacin with a facile, one step synthesis with high yield aiming to improve antibacterial, antifungal and immunological activities. Our methodology allows the introduction of a variety of substituents such as amines, alcohol, phenol, amides and alkyl halides into the core structure of gatifloxacin. The structural modification of gatifloxacin and the rationale for the modification is summarized in Schemes 1 &2. 1,4-dihydroquinoline-3-carboxamide (**4–12**) and 1-4-dihydroquinoline-3-carbohydrazine (**13–14**) analogues of gatifloxacin, formation proceeds via the usual pathway of nucleophilic substitution (Scheme 1). Simplest Fischer method was adopted for the formation of 1,4- dihydroquinoline-3-carboylate derivatives (**15–20**), an example of acid catalyzed esterification (Scheme 2). Our initial success to identify the quinolone based anti-inflammatory agent 7, envisions the potent oxidative burst activity on the whole blood phagocytes, neutrophils and macrophages and has an inhibition effect on T-cell proliferation with almost akin antibacterial efficacy of parent gatifloxacin.

## Results and discussion

### Structural characterization

The absorption intensity of the carbonyl group was decreased and shifted towards right near 1629–1608 cm^–1^ in all the 3-carboxamide derivatives. No peak was observed for carboxylic OH absorption in the FTIR spectra of these derivatives but a distinct strong and un-obscured NH stretch was observed at 3200 cm^-1^. It was observed that the ^1^H NMR spectra of **4–12** derivatives showed a broad singlet of NH of amide at 7.88-5.68 ppm while did not show peak for acidic proton at 12 ppm [16,17]. All these facts clearly indicate that carboxylic site reacted with the selected amines forming amides. FTIR spectra of **15–20** showed the characteristic strong absorption band arising from C=O and C-O; the intensive C=O stretching vibration occurs at higher frequencies 1709–1735 cm^-1^ than C=O of ketone (present in parent drug) due to electron withdrawing effect (inductive effect) indicating formation of ester linkage and successfulness of coupling reaction. The ^1^HNMR spectra of all derivatives do not show signal at 12 ppm, confirming that the carboxylic group of gatifloxacin was utilized in reaction with phenols derivatives. FTIR spectra of **16** and **17** showed C=O stretching at 1729 cm^-1^, the ^1^HNMR spectra as it do not show any signal for NH of amide and a triplet signal of additional aromatic proton attached to C-18 at 7.01 ppm and multiplet signals of protons attached to C −19, 21 at 6.16-6.10 indicate that –OH group of aminophenol utilized in formation of these compound instead of amino group.

### Immunomodulatory effects

Many clinical disorders are associated with immune system. The human immune system comprises of innate and specific immunity. The innate immunity involves a range of specialized cells such as neutrophils, eosinophils and monocyte/macrophages in blood and in many body tissues while the T lymphocytes and B-lymphocytes are two important cells involved in specific immunity [18]. The NADPH oxidase is a multiunit enzyme that is responsible for producing superoxide anion (utilizing oxygen) which is quickly converted to hydrogen peroxide and hydroxyl radicals [19] as an antimicrobial agents. Abnormalities in the constituent peptides of the NADPH oxidase enzyme system lead to dysfunctions characteristic of chronic granulomatous disease (CGD). Neutrophils from CGD defected patients are unable to produce significant oxidative burst following activation. The oxidative burst is impaired in disorders related to innate immunity like in transplant, AIDS patients and in infectious diseases; on the other hand it is highly elevated in inflammatory disorders [20].

**Figure 1:**
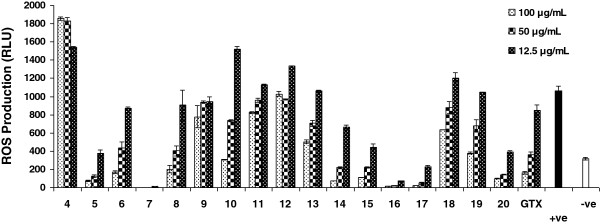
**The Immunomodulatory effects of gatifloxacin (GTX) and its analog compounds on oxidative burst response using whole blood cells.** Each bar represents a mean triplicate reading. +ve = Cells + Zymosan, -ve = Cells without zymosan.

**Figure 2:**
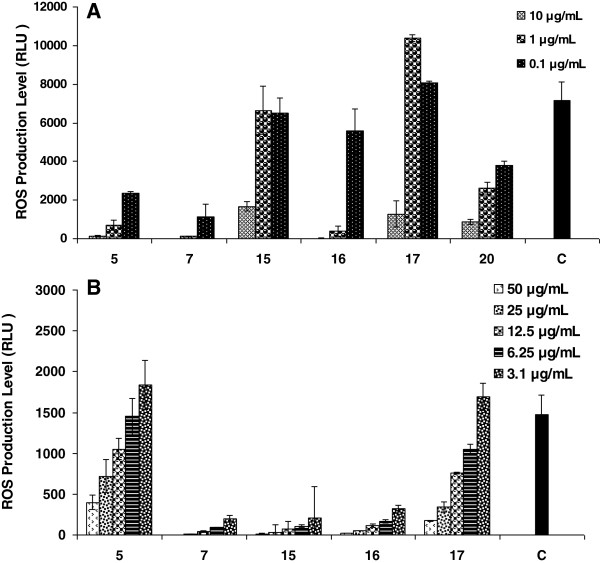
**The Immunomodulatory effects of quinolone derivatives on oxidative burst response using isolated neutrophils (A) and mouse peritoneal macrophages (B).** Each bar represents a mean of triplicate reading after 30-minute incubation and then serum opsonized zymosan activation. The solid bar [black] represents the activated control group cells [Neutrophiles/Macrophages].

**Figure 3:**
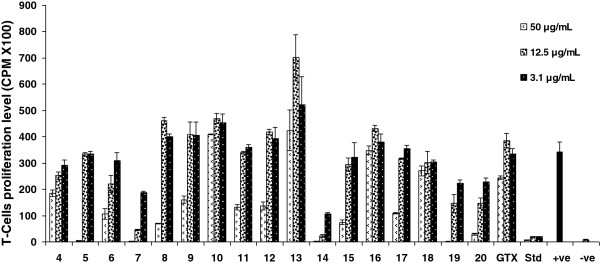
**The modulatory effects of gatifloxacin (GTX) and its analog compounds (4–20) on mitogen activated T-cell proliferation compared with prednisolone (Std).** Each bar represents a mean triplicate reading. +ve = Cells with PHA, -ve = Cells without PHA.

**Figure 4:**
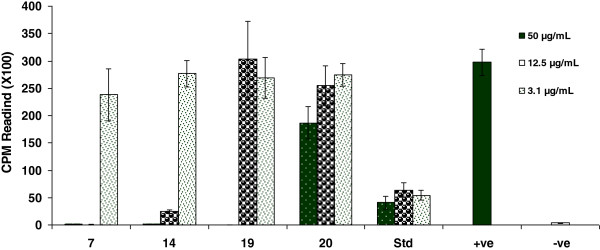
**Represents toxic effect of compounds 5, 7, 14, 19 and 20 on PHA activated T-cells proliferation compared to prednisolone.** T-cells were incubated with compounds/prednisolone for 24 hrs then washed and activated for further 72 hrs with PHA. The bars represent the level of T-cells proliferation compared to controls [cells + PHA (+ve) and cells without PHA (−ve)]. Each bar represents a mean of triplicate reading.

So as to test the immunomodulatory effect of gatifloxacin and its seventeen analogues, we investigated their effect on the oxidative burst ROS production activity of whole blood (Table 1 and Figure 1), isolated neutrophils and macrophages phagocytes (Figure 2) and the inhibitory effects on the proliferation of T cells (Table 2 & Figure 3) along with toxicity studies (Figure 4).

**Table 1:** Screening of gatifloxacin and its derivatives for chemiluminescence activity using whole blood

**Compound**	**RLU Reading**			**Inhibition%**			**IC**_**50**_** ± S.D**
concentration (μg mL^-1^)	100	50	12.5	100	50	12.5	
4	1859.3	1831.4	1540.9	−75.16	−72.53	−45.16	>100
5	74.7	127.7	378.3	92.96	87.97	64.36	<12.5
6	172.8	435.2	873.8	83.72	59	17.68	39.3 ± 12.3
7	−1.7	−1.1	5	100.16	100.1	99.53	<12.5
8	200.5	402.6	905.1	81.11	62.08	14.73	34.6 ± 9.8
9	777.9	939.1	945.2	26.72	11.53	10.96	>100
10	306.9	736.1	1520	71.09	30.66	−43.18	69.6 ± 1.9
11	823.7	958.1	1129	22.41	9.74	−6.39	>100
12	1028.5	970.8	1334	3.11	8.55	−25.72	>100
13	503.6	705.5	1060	52.56	33.54	0.13	90.7 ± 13.2
14	74.3	219.6	663.1	93.01	79.31	37.53	21.5 ± 6.7
15	109.6	222.1	444.6	89.68	79.08	58.12	<12.5
16	15.7	23.1	71.7	98.52	97.82	93.25	<12.5
17	20.5	48.3	226.7	98.07	95.45	78.64	<12.5
18	637.3	879.9	1199.9	39.96	17.11	−13.03	>100
19	379.6	676.6	1047.4	64.24	36.26	1.33	73.6 ± 0.4
20	99.5	143.6	389.8	90.62	86.47	63.28	<12.5
Gatifloxacin	162.4	360.6	852.1	84.7	66.03	19.73	31 ± 0

**Table 2:** Screening of gatifloxacin and its synthesized derivatives using whole blood for their immune modulating inhibitory properties

**Compound**	**CPM Reading**			**Inhibition%**			**IC**_**50**_ **± SD**
Conc (μg/mL)	50	12.5	3.12	50	12.5	3.12	
4	18538.3	25229.4	29076.6	45.86	26.32	15.09	>50
5	476.3	33339.6	33370.4	97.6	−70.7	−70.9	24 ± 0.5
6	10678.5	22012.4	30970.5	45.3	−12.7	−58.6	23.9 ± 9
7	286.6	4554.4	18804.47	99.16	86.7	45.09	3.7 ± 0.1
8	6988.7	46099.7	40114.8	64.2	−136	−105.4	35.5 ± 0.4
9	16087.9	40831.9	40689	17.6	−109.1	−108.3	43.5
10	40893.6	46979.2	45283.2	−109.4	−140.5	−131.8	>50
11	13122.8	33977.4	35965.9	32.8	−74	−84.1	37.5 ±1.5
12	13691.3	41857.5	39169.8	30	−114.3	−100.5	40.7 ± 0.5
13	42380	70222.1	52258.6	−23.7	−105.05	−52.59	>50
14	257.3	2182.5	10766.9	98.7	88.8	44.9	<3.12
15	7470.6	29308.1	32117.4	61.8	−50.1	−64.4	27 ± 2.6
16	34845.8	43188.6	38111.9	−78.4	−121.1	−95.1	>50
17	10892.5	31624.6	35370.22	44.2	−61.9	−81.1	33.0 ± 0.8
18	27012.8	30291.9	30529.1	−38.3	−55.1	−56.3	>50
19	117	14599.9	22362.3	99.4	25.2	−14.5	6.8 ± 0.3
20	2932.7	14825.1	22921.5	85	24	−17.4	8.8 ± 2.8
Gatifloxacin	24450.9	38657.7	33427.3	−25.2	−97.9	−71.1	> 50
Standard	654.2	1906.8	1945.7	98.089	94.432	94.318	< 3.12

ROS can be monitored and quantified by a luminol enhanced chemiluminescence technique. The luminol in a probe detects intracellular ROS after cells activation with serum opsonized zymosan [19,21]. The preliminary screening results of whole blood phagocytosis showed compounds **5, 7, 15, 16, 17** and **20** exhibiting highly potent inhibitory effects on oxidative burst response at all concentrations while gatifloxacin exerted a moderate activity with IC_50_ 31 μg mL^-1^. The% inhibitory activities of these compounds were 64.4, 99.5, 58.1, 93.2, 78.6 and 62.9% respectively at 12.5 μg mL^-1^ concentration (Table 1). In another set of experiment the above mentioned six compounds again demonstrated an extremely potent inhibition on the isolated neutrophils with IC_50_, <0.1, <0.1, 1.7, 0.2, 2.4 and 0.2 μg mL^-1^ respectively (Figure 2A). Compounds **7**, **15** and **16** once more envisaged outstanding activity against macrophages and phagocytes (Figure 2B), compound **7** and **16** having IC_50_ lower than 3.1 μg mL^-1^ while compound **15** and **17** had IC_50_ 8.5 and 12.9 μg mL^-1^ respectively (Table 3).

**Table 3:** **The comparative IC**_**50 **_**(μgmL**^**-1**^**) effect of gatifloxacin and test compounds on oxidative burst of whole blood, isolated PMNs, and mouse peritoneal macrophages**

**Compound**	**Whole blood**	**PMNs**	**Macrophages**
Gatifloxacin	> 30	> 30	> 30
5	<<12.5	<<0.1	24.5 ± 5.6
7	<<12.5	<0.1	<3.1
15	<12.5	1.7 ± 0.6	8.5 ± 0.6
16	<<12.5	0.2 ± 0.1	7.6± 0.6
17	<<12.5	2.4 ± 1.0	12.9 ± 0.2
20	<12.5	0.2 ± 0.1	–

The results of chemiluminescence assay and structures of these compounds suggest that amide and ester derivatives of gatifloxacin with a suitably substituted exocyclic phenyl ring posses potent anti-inflammatory activities. This might be due to the fact that amino group has an ability to be protonated at physiological (pH 7.4) to form ionic, hydrogen, dipole-ionic or dipole–dipole bonding with target molecule which strengthen the pharmacodynamics properties [22]. The compounds having exocyclic substituted phenylenamine ring at C-3 position of the quinolone core showed varying degrees of activities as in case of compounds **5, 7, 15, 16, 17** and **20**. The most active compounds of the series were **7** and **16** that were p-phenylenediamine and m-aminophenol substituted derivatives to fulfill essential structural requirement, described above. However the difference in the activity between the compound **7** and **16** might be due to the presence of additional amino group in compound **7** that afford supplementary binding site in drug molecule. Compounds **16** and **17** are position isomers and **16** is more potent than **17** which demonstrate *m*-amino at phenyl group turn out to exhibit more potent activity than *o*-amino group. The results suggest that the ability of compound to form the phenylamine radical and the stability of this derived radical are important in the anti-inflammatory activities via neutralization of harmful radicals.

Furthermore we tested gatifloxacin and its seventeen derivatives for anti-proliferation effect by measuring the inhibition of phytohemagglutinin (PHA) induced T- cell proliferation by determining radioactive thymidine incorporation and prednisolone was used as standard drug. Results shown in Table 3 & Figure 3 evidently point out that gatifloxacin itself has no suppression effects on T-cells. However compounds **7, 14, 19** and compound **20** exert potent antiproliferative activities. A dose as low as 3.1 μg/mL of compound 14 caused remarkable reduction (68.6%) in T-cell proliferation compared to control. The IC_50_ value was < 3.1 μg mL^-1^ and this activity is not due to toxic effects as 92.9% cells are found alive when tested for toxicity whereas, in case of prednisolone only 34.3% cells remained alive. Compound 7 also envisions its good anti-proliferative T-cell together with excellent ROS oxidative burst effect. It had IC_50_ value 3.7 μg mL^-1^ and 80% cells remained alive in toxicity evaluation assay at 3.1 μg mL^-1^ concentration (Figure 4). Compound **7** and **14** had similar beneficial antiproliferative activity as compared to prednisolone (IC_50_ value < 3.12 μg mL^-1^) but with improved safety profile (Figure 4). Therefore the compound 7 and 14 proved as an excellent quinolone based anti-inflammatory or immunosuppressive activity.

Compounds **19** and **20**, 1,4-dihydroquinoline-3-carboylate analogs, showed significant inhibitory effect on T-cell proliferation with IC_50_ 6.8 μg mL^-1^ and 8.8 μg mL^-1^ respectively (Table 2). The most important is that compound 20 reduced 91.4% T cell proliferation, similar to prednisolone 98.1%. Nevertheless the inhibitory activity of compound **20** is not due to toxic effect since 62.6% cells were found alive whereas in case of predinosolne only 6.9% cells remain alive at 50 μg mL^-1^ (Figure 4). Compound **19** exert 57.4% of immunosuppressive activity without any toxic effect at 12.5 μg mL^-1^ concentration. The results suggest that quinolone based compounds that have exocyclic suitable substituted phenyl ring possess immunosuppressive activity especially when there is phenylhyrazine or phenylenediamine attached to C-3 of quinolone ring envision superb activity with less cytotoxic effect and compound with extra hydroxyl at ortho position of exocyclic phenyl produce significant activity with no toxic effect. However both compounds do not exert any significant effect on oxidative burst of phagocytes.

The structural modification at carboxylic group has resulted in improved anti-inflammatory activities with comparable antibacterial activity to gatifloxacin. Therefore we believe that the C3 structural modifications of gatifloxacin are definitely important in bringing major immunomodulatory changes in these compounds.

## Experimental

### Chemistry

All reagents were of analytical grade purchased from Merck (Germany). Gatifloxacin (98.67%) was gratis from Barrett Hodgson Pakistan. IR and ^1^H-NMR spectra were recorded on Prestige-21 Shimadzu FTIR (KBr) and Bruker AMX (400 MHz), respectively. Chemical shifts are reported in ppm using tetramethylsilane (TMS) as an internal standard. However ^13^C NMR were not performed as gatifloxacin is a well established molecule and structural changes of derivatives were confirmed by IR, ^1^H-NMR and by mass spectra. The mass spectra were recorded on MAT312 Mass spectrometer (Jeol, Tokyo, Japan) operating at 70 eV by electron ionization technique (EI-MS). Zymosan A was purchased from Fluka Biochemika (Switzerland), HBSS^--^ and HBSS^++^ were obtained from Sigma-Aldrich (Steinheim, Germany), Luminol from Alfa Aesar (Karlsruhe, Germany), FBS (Fetal Bovine Serum) purchased from PAA Laboratories GmbH (Pasching, Austria), RPMI-1640 (Roswell Park Memorial Institute) from MP biomedicals (USA), Lyophilized PHA (Phytohemagglutinin) purchased from Sigma–Aldrich (St. Louis, USA). Chemiluminescence and T-Cell proliferation assay were performed using Luminoskan EL, RT, RS from Labsystems (Helsinki, Finland), LS 6500 β-Scintillation Counter from Beckman Coulter (Fullerton, CA, USA) and Cell Harvester System purchased from INOTECH (Dottikon, Switzerland).

### General procedure of synthesis

The carboxamide and carbohydrazide derivatives were prepared as summarized in Scheme 1. 2.48 mmole solution of gatifloxacin in methanol was acidified by adding 1–2 drops of concentrated sulfuric acid and heated at 60°C refluxed for about 4 hours till the consumption of gatifloxacin in ester formation (monitored by TLC). The ester was subjected to nucleophilic attack by adding 3 mmole methanolic solutions of aromatic amines or hydrazine respectively with continuous stirring to generate corresponding carboxamides or carbohydrazides. The reaction was processed for about 2–3 hours till completion, indicated by TLC [23]. The caroboxylates were prepared by stirring the mixture of gatifloxacin (1 gm, 2.48 mmole) and methanolic solution of alcohol or phenol derivative (3 mmole) in acidic medium at 60°C under reflux (Scheme 2). The mixture was cooled to room temperature and excess solvent was removed under reduced pressure; the residue was suspended in water and extracted with ethyl acetate (8 mL x 3). The organic phase was dried over Na_2_SO_4_ (anhydrous), filtered and evaporated to dryness then washed with chloroform and re-crystallized from ethanol-chloroform (3:2) mixture till pure compounds were obtained (ensured by TLC and constant melting point).

#### 1-Cyclopropyl-6-fluoro-8-methoxy-7-(3-methylpiperazin-1-yl)-4-oxo-N-p-tolyl-1,4-dihydroquinoline-3-carboxamide [4]

Yield 78% , m.p. 117°C, UV nm (ε): 221.5 nm (27144), 292.5 nm (12158), 324.5 nm (4590), IR (KBr) ν_max_: 3497, 2960–2852, 1625, 1521, 1443, 1352, 1182, 1043 cm^-1^, ^1^H NMR (MeOD, 400 MHz) δ: 0.94-1.23 (m, 4H, cyclopropane), 1.45-1.47(d, 3H, J = 5.92 CH_3_ of piperazine ring), 1.87-2.25 & 3.46-3.48, m, 7H of piperazine ring), 3.65 (s, 3H, OCH_3_), 3.93-3.95 (m, 1H of cyclopropane), 5.68 or 6.84 (bs, NH of amide), 7.05-7.20 & 7.36-7.629 (m, 4H aromatic), 7.2 (s, MeOD), 7.87-7.89 (d, 1H, J = 11.68), 8.80 (s, 1H). Mass (m/z,%): 464.5(M^+^ 4.6), 409(2.9), 390(3.5), 375(38.1), 319(100), 288(6.3), 245(9), 172(63.5), 77(8) calculated for C_26_H_29_FN_4_O_3_: C, 67.24; H, 6.25; N, 12.06. Found: C, 67.1; H, 6.15; N, 12.04.

#### 1-Cyclopropyl-6-fluoro-8-methoxy-7-(3-methylpiperazin-1-yl)-N-(naphthalen-1-yl)-4-oxo-1,4-dihydroquinoline-3-carboxamide [5]

Yield 71% , m.p. 110°C, UV nm (ε): 214 (73915), 258 (16253), 290 (37988), 324 (12588), IR (KBr) ν_max_: 3470–3350, 3258, 3053, 2857, 2352–2343, 1625, 1579–69, 1456–1442, 1392 cm^-1^, ^1^H NMR (MeOD, 400 MHz) δ: 0.87-0.98 & 1.08-1.30 (m, 4H cyclopropane), 1.37-1.39 (d, 3H, J = 8), 1.53 & 2.86-3.46 (m, 7H, of piperazine ring), 3.74 (s, OCH_3_), 3.97-4.02 (m, 1H, cyclopropane), 6.70-6.75 (dd, 1H, aromatic C31), 7.23 (s, MeOD), 7.26-7.83 (m, 8H, aromatic), 7.88 (d, 1H, NH), 8.79 (s, 1H, aromatic C2). Mass (m/z,%): 500(M^+^ 0.89), 444(9), 56(15.69), 430(1.3), 70(17.02), 375(28.6), 346(2.8), 319(100), 176(4.1), 143(72), 149(5.85) calculated for C_29_H_29_FN_4_O_3_: C, 69.6; H, 5.8; N, 11.2. Found: C, 69.53; H, 5.78; N, 11.19.

#### 1-Cyclopropyl-6-fluoro-8-methoxy-7-(3-methylpiperazin-1-yl)-4-oxo-N-phenyl-1,4-dihydroquinoline-3-carboxamide [6]

Yield 81% , m.p. 198°C, UV nm (ε): 213 (32777), 262 (24724), 313 (23829.39), IR (KBr) ν_max_: 3347, 3291, 3086, 2930, 2860, 2356, 1608, 1550, 1516, 1447, 1245, 1117, 1059 cm^-1^, ^1^H NMR (MeOD, 400 MHz) δ: 0.86-0.98 & 1.06-1.205 (m, 4H cyclopropane), 1.42-1.396 (d, 3H, J = 8), 2.03 (s, 1H, NH), 2.92-3.50 (m, 7H, of piperazine ring), 3.75 (s, OCH_3_), 4.00 (m, 1H, cyclopropane), 6.229 (s, 1H, NH), 7.047-7.11 (m, 1H, aromatic C30), 7.23 (s, MeOD), 7.29-7.36 (m, 2H, aromatic C29,31), 7.83-7.87 (d, 1H, aromatic C5, J =12.17 ), 8.376-8.395 (m, 2H, C28,32 ), 8.791 (s, 1H, aromatic C2). Mass (m/z,%): 450(M^+^ 1.3), 373(8.4), 319(100), 291(100), 276(13.4), 247(9.2), 219(9.6), 188(3.1), 134(2.6), 115(1.5) calculated for C_25_H_27_FN_4_O_3_: C, 66.66; H, 6.00; N, 14.22. Found: C, 66.21; H, 6.12; N, 14.18.

#### N-(3-Aminophenyl)-1-cyclopropyl-6-fluoro-8-methoxy-7-(3-methylpiperazin-1-yl)-4-oxo-1,4-dihydroquinoline-3-carboxamide [7]

Yield 85% , m.p. 130°C, UV nm (ε): 212 (25790), 292 (16538), 325 (6058), 464 (14048), IR (KBr) ν_max_: 3387, 3208, 3063, 2971, 2863, 2382, 1610, 1510, 1469, 1283, 1220, 1138, 1075, 893 cm^-1^, ^1^H NMR (MeOD, 400 MHz) δ: 0.96-0.98 & 1.08-1.20 (m, 4H cyclopropane), 2.89-3.40 (m, 10H, of piperazine ring), 3.75 (s, OCH3), 3.96-4.02 (m, 1H, cyclopropane), 5.68 (s, 1H, NH), 6.87-6.92 (m, 10H, aromatic), 7.2 (s, MeOD), 7.8-7.87 (d, 1H, aromatic C5, J =12), 8.75 (s, 1H, aromatic C2). Mass (m/z,%): 465(M^+^ 1.39), 373(1.6), 319(100), 275(22.8), 219(5.8), 189(3.9), 147(2.0), 95(4.6), 76(5.0), 70(31), 56(13) calculated for C_25_H_28_FN_5_O_3_: C, 64.51; H, 6.02; N, 15.05. Found: C, 64.50; H, 6.02; N, 15.1.

#### 1-(1-Cyclopropyl-6-fluoro-8-methoxy-7-(3-methylpiperazin-1-yl)-4-oxo-1,4-dihydroquinoline-3-carbonyl)urea [8]

Yield 79% , m.p. 152°C, UV nm (ε): 213.5 (21102), 232 (21849), 293.5 (40328), 324.5 (14382), IR (KBr) ν_max_: 3440, 2978, 2862, 2480, 2382–2364, 1627, 1450, 1396, 1286, 1215, 1064, 1002.31, 829, 736 cm^-1^, ^1^H NMR (MeOD, 400 MHz) δ: 0.94-0.98 & 1.11-1.20 (m, 4H cyclopropane), 1.36-1.38 (d, 3H, J = 6.69), 1.97 (s, 1H, NH), 2.90-3.41 (m, 8H, of piperazine ring), 3.75 (s, OCH_3_), 3.96-4.03 (m, 1H, cyclopropane), 4.51 (s, 1H, NH), 7.24 (s, MeOD), 7.82-7.86 (d, 1H, aromatic C5, J =12), 8.78 (s, 1H, aromatic C2). Mass (m/z,%): 417(M^+^ 4.4), 362(2.3), 319(51), 275(100), 221(3.5), 167(2.5), 137(14), 95(6.64), 76(3) calculated for C_20_H_24_FN_5_O_4_: C, 57.55; H, 5.75; N, 16.78. Found: C, 57.51; H, 5.68; N, 16.74.

#### 2-(1-Cyclopropyl-6-fluoro-8-methoxy-7-(3-methylpiperazin-1-yl)-4-oxo-1,4-dihydroquinoline-3-carboxamido)benzoic acid [9]

Yield 80% , m.p. 250°C, UV nm (ε): 228.5 (31597), 292.5 (37924), 325.5 (13728), IR (KBr) ν_max_: 3413, 3304, 2853, 1726, 1621.7, 1513.69, 1426.09, 1369.57, 1278, 1056, 1034, 926.6, 804, 760.8, 673.91 cm^-1^, ^1^H NMR (MeOD, 400 MHz) δ: 0.92-0.93 & 1.13-1.24 (m, 4H cyclopropane), 1.25-1.27 (d, 3H, J = 8), 3.10-3.45 (m, 7H, of piperazine ring), 3.66 (s, OCH_3_), 3.94-3.98 (m, 1H, cyclopropane), 6.53-6.59 & 7.15-7.18 (m, 4H, aromatic) 7.2 (s, MeOD), 7.78-7.81 (d, 1H, aromatic C5), 8.74 (s, 1H, aromatic C2). Mass (m/z,%): 494(M^+^ 0.59), 450(2), 373(3.7), 319(58), 263(1.4), 233(1.5), 190(2.4), 137(72.8), 119(100), 76(2.0) calculated for C_26_H_27_FN_4_O_5_: C, 63.15; H, 5.46; N, 11.33. Found: C, 63.12; H, 5.64; N, 11.30.

#### 5-Benzamido-1-cyclopropyl-6-fluoro-8-methoxy-7-(3-methylpiperazin-1-yl)-4-oxo-1,4-dihydroquinoline-3-carboxylic acid [10]

Yield 69% , m.p. 92-93°C, UV nm (ε): 230 (26840), 293 (35577), 326 (12660), 693 (1228), IR (KBr) ν_max_: 3409, 3051, 2863, 2480, 2374, 1612, 1575, 1460–1446, 1392,-61, 1321, 1281, 1053.86, cm^-1^, ^1^H NMR (MeOD, 400 MHz) δ: 0.96-0.98 & 1.09-1.23 (m, 4H cyclopropane), 1.57, 2.87-3.3 (m, 8H, of piperazine ring), 3.75 (s, OCH_3_), 3.97-4.02 (m, 1H, cyclopropane), 5.8-6.1 (bs, 1H, NH), 7.23 (s, MeOD), 7.41-7.51 & 7.77-7.89 (m, 5H, aromatic), 8.71 (s, 1H, aromatic C_2_). Mass (m/z,%): 478(M^+^ 1.39), 373(28), 319(100), 275(20), 219(5.2), 167(3.4), 125(3.4), 95(6.5), 76(3.68), 70(22), 56(18) calculated for C_26_H_27_FN_4_O_4_: C, 65.27; H, 5.64; N, 11.71. Found: C, 65.12; H, 5.60; N, 11.69.

#### 1-Cyclopropyl-N-ethanethioyl-6-fluoro-8-methoxy-7-(3-methylpiperazin-1-yl)-4-oxo-1,4-dihydroquinoline-3-carboxamide [11]

Yield 75% , m.p. 143°C, UV nm (ε): 208 (53093), 232 (66000), 283 (71517), IR (KBr) ν_max_: 3433, 2987, 2847, 2480, 2183, 1629, 1463, 1323, 1284, 1057, 1005, 940, 892, 826 cm^-1^, ^1^H NMR (MeOD, 400 MHz) δ: 0.91-0.96 & 1.14-1.19 (m, 4H cyclopropane), 1.37-1.38 (d, 3H, J = 5.42), 1.90 (s, 3H, C20), 3.19-3.54 (m, 7H, of piperazine ring), 3.75 (s, OCH_3_), 3.94-3.99 (m, 1H, cyclopropane), 7.2 (s, MeOD), 7.61 (d, 1H, aromatic C5, J =12 ), 8.75 (s, 1H, aromatic C2). Mass (m/z,%): 432(M^+^ 0.99), 376(8.38), 319(100), 233(4.6), 179(68), 126(42), 96(24), 77(30), 70(20), 56 (24) calculated for C_21_H_25_FN_4_O_3_S: C, 58.33; H, 5.78; N, 12.96. Found: C, 58.31; H, 5.74; N, 12.94.

#### 1-Cyclopropyl-6-fluoro-8-methoxy-7-(3-methylpiperazin-1-yl)-4-oxo-N,N-diphenyl-1,4-dihydroquinoline-3-carboxamide [12]

Yield 89% , m.p. 132°C, UV nm (ε): 206 (50852), 290 (22344), IR (KBr) ν_max_: 3417, 3070, 2850, 2382–40, 1619, 1462, 1360, 1282, 1208, 1172, 1147, 1140, 1059, 995.71, 935.8 cm^-1^, ^1^H NMR (MeOD, 400 MHz) δ: 0.96-0.98 & 1.08-1.20 (m, 4H, cyclopropane), 2.89-3.40 (m, 10H, of piperazine ring), 3.75 (s, OCH_3_), 3.96-4.02 (m, 1H, cyclopropane), 5.68, 6.87-6.92 (m, 10H, aromatic), 7.23 (s, MeOD), 7.87 (d, 1H, J = 12), 8.78(s,1H). Mass (m/z,%): 526(M^+^ 0.32), 428(2.8), 374(100), 344(36), 176(2.8) calculated for C_31_H_31_FN_4_O_3_: C, 70.72; H, 5.89; N, 10.64. Found: C, 70.71; H, 5.83; N, 10.52.

#### 1-Cyclopropyl-6-fluoro-8-methoxy-7-(3-methylpiperazin-1-yl)-4-oxo-1,4-dihydroquinoline-3-carbohydrazide [13]

Yield 72% , m.p. 250°C, UV nm (ε): 212.5 (18321), 231.5 (17004), 293 (31039), 325 (11179), IR (KBr) ν_max_: 3445, 3155.9, 2951, 2871, 2497, 1623, 1516, 1467, 1250, 1104, 1059–1094, 944 cm^-1^, ^1^H NMR (MeOD, 400 MHz) δ: 0.95-1.19 (m, 4H cyclopropane), 1.43-1.45 (d, 3H, J = 8), 2.38 (s, 3H, NH), 3.20-3.76 (m, 7H, of piperazine ring), 3.71 (s, OCH_3_), 3.97-3.99 (m, 1H, cyclopropane), 7.24 (s, MeOD), 7.83-7.87 (d, 1H, J = 12), 8.78 (s, 1H). Mass (m/z,%): 489(M^+^ 11.2), 373(9.9), 319(100), 288(4.9), 259(18.4), 244(3.7), 216(3.2), 188(2.5), 146(1.5), 92(1.2) calculated for C_19_H_24_FN_5_O_3_: C, 58.60; H, 6.21; N, 17.98. Found: C, 58.59; H, 6.17; N, 17.95.

#### 1-Cyclopropyl-6-fluoro-8-methoxy-7-(3-methylpiperazin-1-yl)-4-oxo-N-phenyl-1,4-dihydroquinoline-3-carbohydrazide [14]

Yield 75% , m.p. 170°C, UV nm (ε): 213 (28942), 230 (27927), 291.5 (44364), 325.5 (17457), IR (KBr) ν_max_: 3433, 3083, 2849, 2483, 2383, 1621, 1522, 1468–47, 1060, 932 cm^-1^, ^1^H NMR (MeOD, 400 MHz) δ: 0.87-1.39 (m, 4H cyclopropane), 1.46-1.48 (d, 3H, J = 8), 1.97 (s, 1H, NH), 2.40 & 3.00-3.39 (m, 7H, of piperazine ring), 3.73 (s, OCH_3_), 3.95-4.01 (m, 1H, cyclopropane), 6.80-6.82 (m, 2H, aromatic C_19,23_), 6.89-6.91 (m, 1H, aromatic C_21_), 7.24 (s, MeOD), 7.42-7.51 (m, 2H, aromatic C_20,22_), 7.50 (d, 1H, aromatic C_5_, J =12 ), 8.312 (s, 1H, C = N – NH -), 8.76 (s, 1H, aromatic C_2_). Mass (m/z,%): 465.5(M^+^ 0.49), 388(1.07), 373(4.6), 319(100), 288(9.2), 259(28.1), 219(13.4), 188(5), 148(3), 92(5) calculated for C_25_H_28_FN_5_O_3_: C, 64.50; H, 6.06; N, 15.04. Found: C, 64.48; H, 6.05; N, 15.01.

#### Phenyl1-cyclopropyl-6-fluoro-8-methoxy-7-(3-methylpiperazin-1-yl)-4-oxo-1,4-dihydroquinoline-3-carboxylate [15]

Yield 75% , m.p. 156°C, UV nm (ε): 211 (21855), 292 (28415), 325 (10129), IR (KBr) ν_max_: 3065, 2859, 2362, 1725, 1618, 1469–1458, 1319, 1273, 1209, 1059 cm^-1^, ^1^H NMR (MeOD, 400 MHz) δ: 0.889-0.931 & 1.01-1.03 (m, 4H of cyclopropane, 1.12-1.14(d, 3H of CH_3_ of piperazine ring, J = 8), 2.79-3.34 (m, 8H of piperazine), 3.68 (s, 3H, OCH_3_), 3.94-3.97 (m, 1H, of cyclopropane), 6.70-6.75 (m, 4H aromatic), 7.07-7.11 (m, 4H, aromatic), 7.07-7.11 (t, 1H, aromatic J = 7.88), 7.23 (s, MeOD), 7.73-7.76 (d, 1H aromatic, J = 12), 8.70 (s, 1H). Mass (m/z,%): 451(M^+^ 0.97), 395(1.6), 365(27.69), 351(6.22), 323(100), 230(3.39), 176(2.76), 70(10.43), 56(10.20) calculated for C_25_H_26_FN_3_O_4_: C, 66.51; H, 5.80; N, 9.31. Found: C, 66.31; H, 5.80; N, 9.28.

#### 3-Aminophenyl-1-cyclopropyl-6-fluoro-8-methoxy-7-(3-methylpiperazin-1-yl)-4-oxo-1,4-dihydroquinoline-3-carboxylate [23]

Yield 73% , m.p. 140-142°C, UV nm (ε): 214 (21338), 231 (21410), 292 (39853), 325 (14058), IR (KBr) νmax: 3443, 3034, 2834, 1721, 1600, 1569.57, 1465.22, 1365.22, 1260.87, 1169, 1134, 1034, cm^-1^, ^1^H NMR (MeOD, 400 MHz) δ: 0.897- 0.937 & 1.13-1.14 (m, 4H, cyclopropane), 1.31-1.33 (d, 1H, J = 8, CH_3_ of piperazine), 2.991 (s, 3H, NH), 3.01-3.15 (m, 7H of piperazine), 3.69 (s, 1H, OCH_3_), 3.93-3.98 (m, 1H, cyclopropane), 6.10-6.16 (m, 2H aromatic, C_19-21_), 6.83 (m, 1H, aromatic C_17_), 7.01 (t, 1H, C_18_), 7.23 (MeOD), 7.57 (d, 1H, J = 12), 8.71 (s, 1H). Mass (m/z,%): 466(M^+^ 0.49), 374(3.34), 319(100), 275(11.7), 219(5.84), 166(3.28), 136(3.19), 70(43), 56(27.47) calculated for C_25_H_27_FN_4_O_4_: C, 64.37; H, 5.83; N, 12.01. Found: C, 64.36; H, 5.87; N, 12.0.

#### 2-Aminophenyl-1-cyclopropyl-6-fluoro-8-methoxy-7-(3-methylpiperazin-1-yl)-4-oxo-1,4-dihydroquinoline-3-carboxylate [24]

Yield 79%, m.p. 165°C, UV nm (ε): 213 (22304), 232 (22727), 293.5 (41090), 324 (14722). IR (KBr) ν_max_: 3424–3319, 3013.19, 2856, 1725.21, 1629, 1537–1515, 1450.431, 1376, 1315–1254, 1057 cm^-1^, ^1^H NMR (MeOD, 400 MHz) δ: 0.94-1.23 (m, 4H cyclopropane), 2.89 (s, 3H, NH), 2.92-3.4 (m, 7H, of piperazine ring), 3.74 (s, OCH_3_), 3.97-4.02 (m, 1H, cyclopropane), 6.73 (m, 4H aromatic), 7.83-7.87 (d, 1H, J = 16), 8.79 (s, 1H). Mass (m/z,%): 466(M^+^ 1.2), 374(21.3), 319(100), 275(27.06), 219(3.3), 166(3.6), 136(7), 95(25.25), 70(41.5), 56(51.9) calculated for C_25_H_27_FN_4_O_4_: C, 64.37; H, 5.83; N, 12.01. Found: C, 64.35; H, 5.86; N, 12.0.

#### 3-Hydroxyphenyl-1-cyclopropyl-6-fluoro-8-methoxy-7-(3-methylpiperazin-1-yl)-4-oxo-1,4-dihydroquinoline-3-carboxylate [25]

Yield 70%, m.p. 165.5°C, UV nm (ε): 214 (26267), 292 (32648), 325 (11685). IR (KBr) ν_max_: 3428, 2990, 2841, 2742, 2344, 1735, 1619, 1577, 1543, 1460, 1450, 1395, 1365, 1280, 1215, 1180, 1143, 1065–1058, 998, 938 cm^-1^, ^1^H NMR (MeOD, 400 MHz) δ: 0.90-0.94 & 1.12-1.18 (m, 4H of cyclopropane, 1.372-1.389(d, 3H of CH_3_ of piperazine ring, J = 6.8), 3.02-3.44 (m, 8H of piperazine), 3.70 (s, 3H, OCH_3_), 3.94-3.99 (m, 1H, of cyclopropane ), 6.21-6.26 (t, 1H aromatic, C_20_ J = 16 ), 7.2 (s, MeOD), 7.80-7.77 (d, 1H, aromatic J = 12), 8.73 (s, 1H). Mass (m/z,%): 467(M^+^ 0.3), 374(25.93), 319(100), 275(13.9), 219(6.08), 70(44), 56(39.5) calculated for C_25_H_26_FN_3_O_5_: C, 64.23; H, 5.61; N, 8.99. Found: C, 64.22; H, 5.60; N, 8.78.

#### 3,5-Dihydroxyphenyl-1-cyclopropyl-6-fluoro-8-methoxy-7-(3-methylpiperazin-1-yl)-4-oxo-1,4-dihydroquinoline-3-carboxylate [26]

Yield 72%, m.p. 75-80°C, UV nm (ε): 207 (31605), 292 (24701). IR (KBr) νmax: 3433, 2978, 2935, 2873, 2402, 1733–1723, 1621, 1455, 1438, 1276, 1122, 1070 cm^-1^, ^1^H NMR (MeOD, 400 MHz) δ: 0.76-0.91 & 1.14-1.36 (m, 4H of cyclopropane, 1.47(d, 3H of CH_3_ of piperazine ring, J = 8), 3.24-3.45 (m, 8H of piperazine), 3.62 (s, 3H, OCH_3_), 4.05-4.14 (m, 1H, of cyclopropane ), 6.15 (d, 2H aromatic, J = 9.04 ), 6.37 (t, 2H, J = 16), 7.23 (s, MeOD), 7.31 (s, 2H, OH), 7.76 (d, 1H, aromatic J = 12), 8.73 (s, 1H). Mass (m/z,%): 483(M^+^ 0.59), 374(1.9), 319(100), 275(16.18), 219(2.8), 76(24), 70(27), 56(9.89) calculated for C_25_H_26_FN_3_O_6_: C, 62.10; H, 5.42; N, 8.69. Found: C, 62.09; H, 5.41; N, 8.68.

#### 2-Hydroxyphenyl-1-cyclopropyl-6-fluoro-8-methoxy-7-(3-methylpiperazin-1-yl)-4-oxo-1,4-dihydroquinoline-3-carboxylate [16]

Yield 78%, m.p. 82°C, UV nm (ε): 214 (26358), 292(31965), 325 (10685). IR (KBr) νmax: 3250, 2991, 2856, 1709, 1695, 1565, 1513, 1491, 1382, 1278.26, 1182.6, 1104 cm^-1^, ^1^H NMR (MeOD, 400 MHz) δ: 0.90-0.94 & 1.12-1.18 (m, 4H of cyclopropane, 1.372-1.389(d, 3H of CH_3_ of piperazine ring, J = 6.8), 3.02-3.44 (m, 8H of piperazine), 3.70 (s, 3H, OCH_3_), 3.94-3.99 (m, 1H, of cyclopropane ), 6.21-6.26 (t, 1H aromatic, C_20_ J = 16 ), 7.2 (s, MeOD), 7.80-7.77 (d, 1H, aromatic J = 12), 8.73 (s, 1H). Mass (m/z,%): 467(M^+^ 3.3), 374(38), 319(100), 275(4.6), 219(2.8), 189(5.6), 147(9.1), 95(3), 76(34), 70(28), 56(50) calculated for C_25_H_26_FN_3_O_5_: C, 64.23; H, 5.61; N, 8.99. Found: C, 64.19; H, 5.60; N, 8.98.

## Biological studies

### Phagocyte chemiluminescence

#### Preparation of opsonized zymosan and luminol

The opsonization of zymosan particles was carried out following Wick’s method [24] with some modifications. Briefly, zymosan (100 mg) was mixed in 5 mL phosphate buffer saline (PBS) pH 7.4 and 5 ml fresh-pooled serum from healthy human volunteers. The mixture was incubated at 37°C in a shaking water bath for 30 min, then centrifuged, washed twice with PBS and finally re-suspended in 5 ml of PBS. The mixture was stored at −20° C till use and was brought to room temperature immediately before experiment. Luminol solution (7 × 10^-5^ M) was prepared by dissolving 1.8 mg of luminol in 1 mL borate buffer pH 9.1 and vortexed for 5–10 min. The solution was then further diluted up to 10 mL with Hanks balanced salt solution HBSS^++^ (containing Ca and Mg), to give 180 μg mL^-1^.

### Isolation of polymorphoneutrophils (PMNs) and mouse peritoneal macrophages

The heparinized human whole blood was diluted with Hanks balanced salt solution (HBSS^--^) and mixed with one third of dextrin (3% in 0.9% NaCl) solution for differential sedimentation and removal of erythrocytes. After gentle mixing, it was kept at room temperature for 20 min undisturbed. The upper layer containing leucocytes was collected and gently layered over an equal volume of lymphocytes separation medium (LSM) and then subjected to centrifugation at 400 g for 25 min at room temperature. After removal of the upper phase, neutrophils were collected and subjected to hypotonic lysis of RBC with sterile distilled water for one minute, and then washed twice with HBSS^--^. Macrophages from mouse peritoneal cavity were obtained as described in our previous paper [23]. Cells were re-suspended in HBSS^--^ to give 1 × 10^6^ /mL.

### Chemiluminescence assay

The assay was performed as described by Helfand et al., [25] protocol. Briefly gatifloxacin and the other derivatized compounds were diluted in three different concentrations in HBSS^++^ containing calcium and magnesium. 25 μL of either diluted blood (1:50 dilution in sterile PBS, pH 7.4) or polymorphoneutrophils (1 x 10^6^ mL^-1^) or mouse peritoneal macrophages (1 × 10^6^ mL^-1^) were added to the culture reaction. After 15 minutes of incubation for whole blood and 30 minutes for isolated cells, 25 μL (7 × 10^-5^ M) luminol, followed by 25 μL (20 mg mL^-1^) serum opsonized zymosan-A. HBSS^++^ alone without compounds was run as negative control. The level of reactive oxygen species (ROS) after incubation with compounds was monitored for 50 minutes by the Luminometer. The total ROS level was recorded as total light produced and recorded during the 50 minutes scan. The total integral chemiluminescence reading was expressed in the relatively light unit (RLU).

### T-cell proliferation assay

*In vitro* cell proliferation assay was carried out using H^3^ thymidine incorporation method based on technique of Nielsen et al., [26] in a sterile environment. Peripheral blood mononuclear cells (PBMC) were isolated from heparinized venous blood of healthy human by Ficoll-Hypaque gradient centrifugation. These cells cultured in a 96 well round bottom plate at concentration of 2 x 10^6^ cells mL^-1^ in RPMI-1640 media supplemented with 5% FBS in presence of compounds and Phytohemagglutinin (PHA) with concentration of 5 μg mL^-1^. The concentrations of compounds were 3.1, 12.5 and 50 μg mL^-1^, each used in triplicate. The plate was incubated at 37°C for 72 hrs in 5% CO_2_ incubator with the final volume of 0.2 mL per well. After 72 hrs, 25 μl of H^3^ thymidine was (0.5μci/well) added to the culture plate incubated for further 18 hrs. Cells were harvested on a glass fiber filter using cell harvester system. Effect of compounds on proliferation of cells was measured quantitatively by liquid β-Scintillation Counter. Results were recorded as count per minute (CPM). Inhibitory effect of compounds was calculated in comparison to the control.

### Toxicity on T cells

The toxicity of compounds showing inhibitory effect on T cells was also analyzed using same procedure of T cell proliferation. Cells were cultured in presence of compounds for 24 hrs and after one day compounds were removed by washing cells before addition of the PHA (5 μg/mg mL^-1^) for 72 hrs. After 72 hrs, 25 μl of radioactive H^3^ thymidine was added to each well in the plate for further 18 hrs. Results were recorded as count per minute (CPM) using the liquid scintillation counter.

## Conclusion

Seventeen derivatives of gatifloxacin were synthesized, characterized and tested for immunomodulatory activities in phagocyte chemiluminescence and T-Cell proliferation assay. The anti-inflammatory mechanism was elucidated, which provided valuable information for further studies on the novel anti-inflammatory quinolones. The most active quinolone compounds had IC_50_ values < 3.1 μg mL^-1^, while several derivatives were not active at a concentration of 100 μg mL^-1^. In SAR studies, the data suggested that C-3 of quinolone ring with exocyclic substituted phenylenamine ring influenced the immunomodulatory activities. In particular, p-phenylendiamine substituted analog 7 and m-aminophenol substituted analogue **16** exhibited highly suppressive oxidative burst activity of neutrophils, macrophages and phagocytes with significant antibacterial activity compared to that of gatifloxacin. These studies also demonstrated that exocyclic phenyl ring suitably substituted with hydroxyl, amino or hydrazide group envision potent inhibitory effect on cell immediate immunity as compared to humoral immunity with reduced cytotoxic effect especially phenylhydrazide and phenyl hydroxyl analogs. Currently the intensive studies on compounds **7, 16, 14** and **20** including the detailed structure activity relationship and the anti-inflammatory mechanism are in progress.

## Competing interests

The authors declare that they have no competing interest.

## Authors’ contributions

NS conceived of the study, and participated in the synthesis of derivatives. MSA participated in its SAR studies and helped to draft the manuscript. AN carried out the synthesis, purification and characterization of the compounds. AM carried out biological screening studies. All authors have read and approved the final manuscript.
